# Case report: Biliobronchial fistula after biliary tract stenosis

**DOI:** 10.3389/fmed.2022.1075745

**Published:** 2022-12-16

**Authors:** Luís Maurício Batalin Júnior, Mariana Conceição e Silva Seleme Zandoná, Thomaz Almeida Vargas, Julio Cesar de Oliveira, Juliana Rocha Souza Chiappetto, Cassio Vieira Oliveira, Fernando Gomes Romeiro, Suzana Erico Tanni

**Affiliations:** ^1^Pulmonary Division of Internal Medicine, Botucatu School of Medicine—UNESP, São Paulo, Brazil; ^2^Gastroenterology Division of Internal Medicine, Botucatu School of Medicine—UNESP, São Paulo, Brazil

**Keywords:** biliobronchial fistula, biliary tract stenosis, bilioptysis, cyclic jaundice, pneumonia

## Abstract

Biliobronchial fistula (BBF) is a rare abnormality resulting from congenital or acquired communication between the bile ducts and the bronchial tree. Patients often suffer from chronic cough, dyspnea, and bilioptysis, a pathognomonic symptom of this condition. Conservative methods such as less-invasive procedures are gradually consolidating. Nonetheless, surgery remains the primary treatment, especially in more complex cases. We present the case of a 44-year-old woman with a chronic cough, no verified periods of fever, cyclic jaundice, and episodes of yellowish sputum. She had undergone cholecystectomy in 2018 and had been hospitalized several times since for pneumonia treatment. All consequent investigations for mycobacteriosis were negative. When referred to our hospital, she had cyclic jaundice and parenchymal consolidation in the right lower lobe. Suspected bilioptysis motivated the search for a biliobronchial fistula. Magnetic resonance cholangiography (MRC) confirmed stenosis of the biliary tract and fistulous path, and sputum analysis indicated high bilirubin levels. External biliary bypass was performed as an initial conservative and definitive therapy due to the presence of liver cirrhosis. Although BBF is a rare condition when bilioptysis is suspected, a diagnostic investigation should be initiated. Our case study proposes two criteria for diagnosis: an imaging exam demonstrating the fistulous path and confirmation of bilirubin in the sputum or bronchoalveolar lavage (BAL). When diagnosed, surgical correction should be performed.

## Introduction

Peacock first described biliobronchial fistula (BBF) in 1850 as a rare abnormality resulting from the pathological communication between the bile ducts and the bronchial tree (1). Its underlying cause can be congenital or acquired, related to trauma, liver infection, neoplasia, or biliary obstruction (2–7).

Patients with BBF usually present bile expectoration as a cardinal symptom. Other clinical signs include cough, dyspnea, and abdominal or thoracic pain. The diagnosis is based on clinical symptoms and confirmation of bile in the sputum or bronchoalveolar lavage. Less-invasive procedures can be attempted, but conservative surgery is still the primary treatment, especially for more complex cases. However, there is no established guideline for the optimal management of this rare condition, and treatment must be individualized.

## Case report

We report the clinical scenario of a 44-year-old woman who presented to our hospital with a 4-year history of cough, no verified periods of fever, cyclic jaundice, and expectoration of yellowish sputum. The patient had undergone cholecystectomy in 2018 and was hospitalized several times for the treatment of bacterial pneumonia as she started complaining of cyclic jaundice and frequent cough with dyspnea after surgery.

Her chronic cough during those hospitalizations led to several sputum culture analyses, including mycobacteriosis, which was negative. Concomitantly, cirrhosis diagnosis was performed due to bile duct stenosis using magnetic resonance cholangiography (MRC) and she was classified with a MELD (model for end-stage liver disease) score of 17 and a Child–Pugh score of 7.

In a previous hospital admission, the general examination identified a regular general condition with severe jaundice. Systemic examination showed thoracic auscultation with a decreased vesicular murmur, mainly at the lower right hemithorax, associated with crackling noises. The liver was palpable at 2 cm below the right costal margin with a blunt edge and a previous cholecystectomy scar.

**Figure 1:**
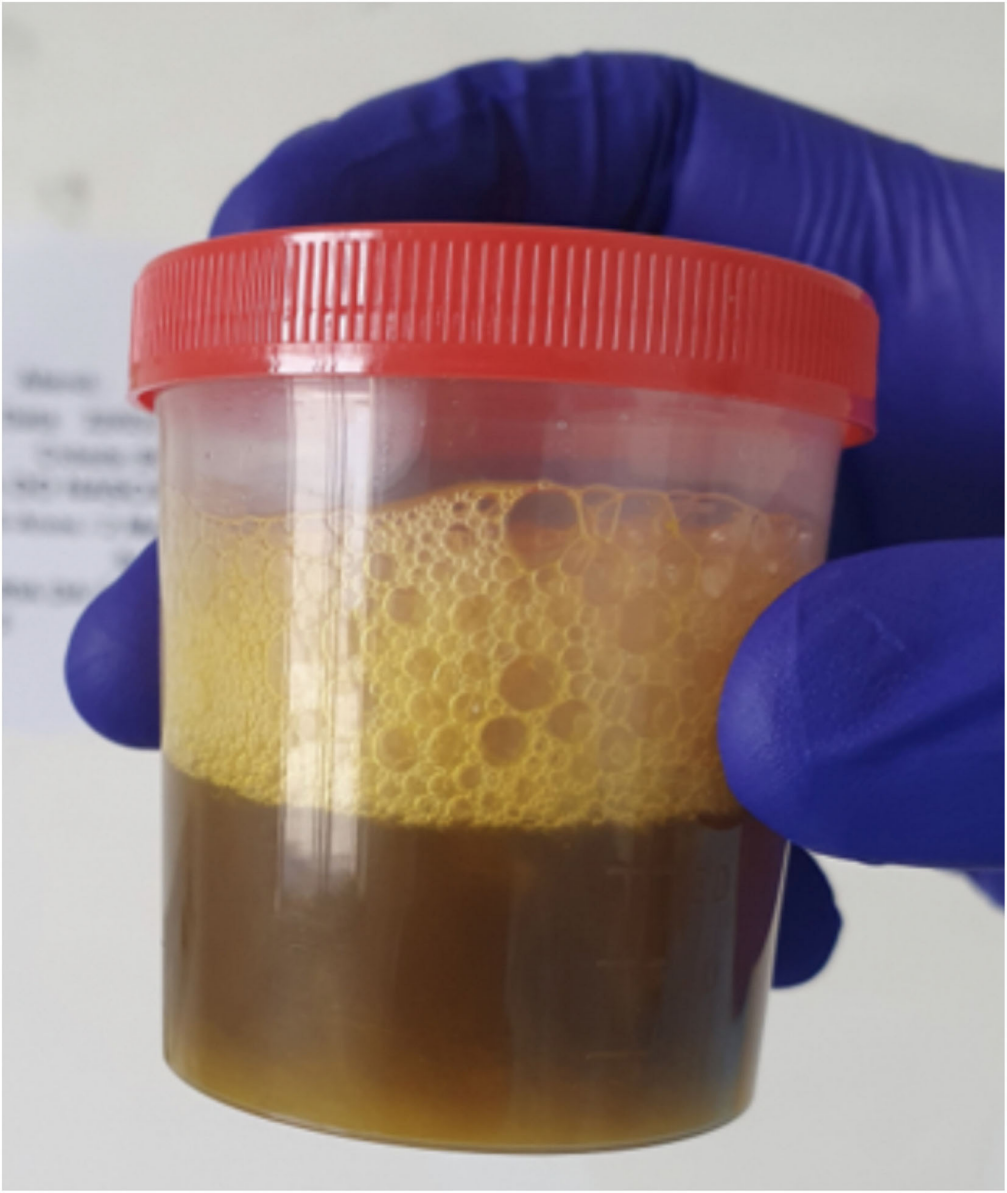
Sample of yellowish expectoration.

During the current hospital admission, the patient presented with a cough with yellowish expectorant (Figure 1), dyspnea, and fever and was started on antibiotics for pneumonia. Initial laboratory tests showed compromised liver function (total bilirubin = 8.6 mg/dL, conjugate bilirubin = 7.2 mg/dL) with signs of acute liver injury (AST = 218 U/L, ALT = 131 U/L, GGT = 533 U/L, AP = 1,301 U/L). Blood count presented with hemoglobin = 14.2 g/dL, hematocrit = 42.8%, white blood cells = 10.9 × 103/mm3, and platelets 343 × 103/mm3, without abnormalities in the coagulogram.

**Figure 2:**
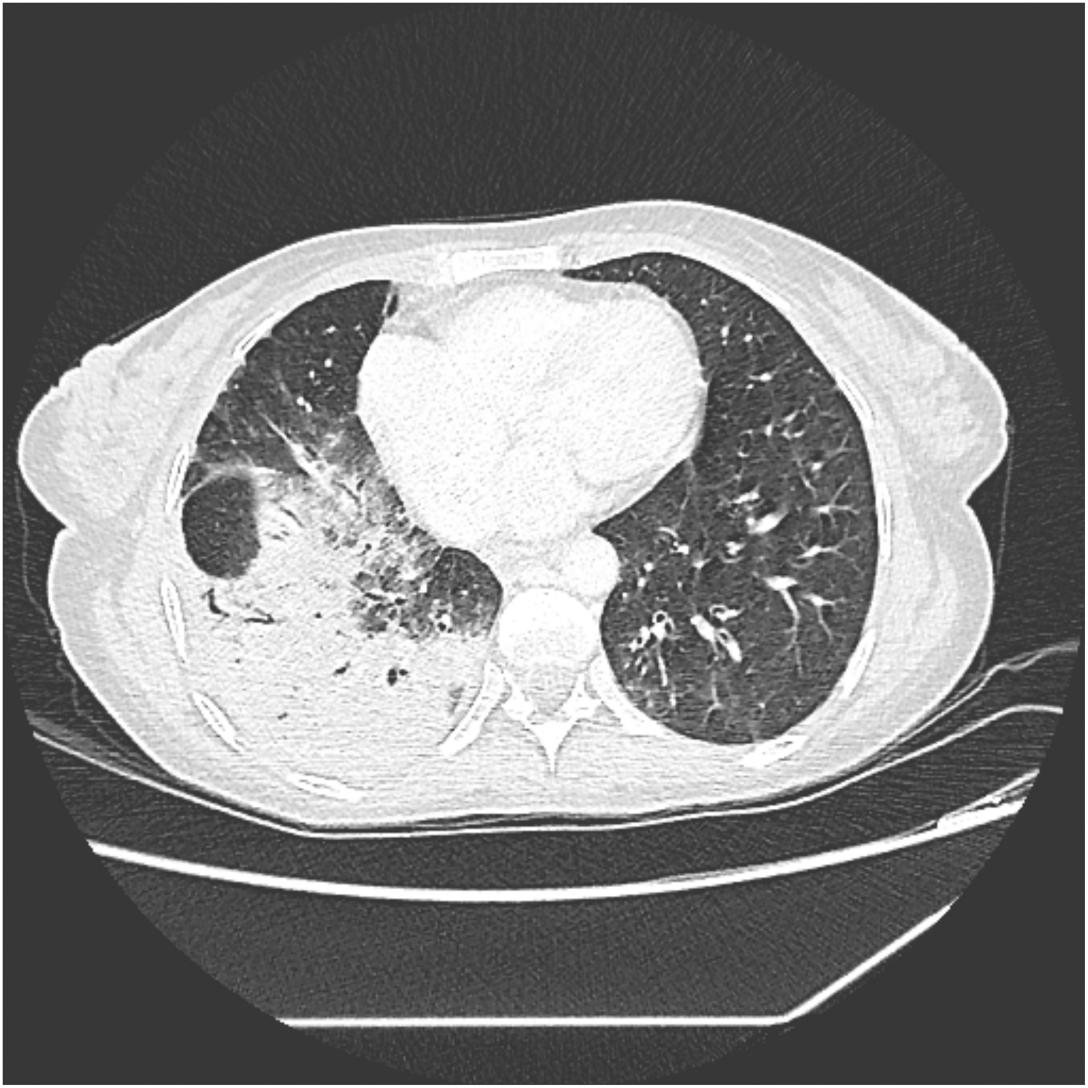
Slice of CT scan showing parenchymal consolidation of the right lower lobe.

Chest and abdominal computed tomography (CT) showed a right lower lobe alveolar consolidation with air bronchograms, surrounded by opacities with ground-glass attenuation compatible with an inflammatory or infectious process (Figure 2). Moreover, the liver showed an enlarged right lobe, with chronic disease characteristics and marked dilation of the intrahepatic bile ducts, without an obstructive factor or dilatation of the hepatocholedochal duct. A biochemical analysis of the sputum showed increased conjugated bilirubin (14.4 mg/dL).

**Figure 3:**
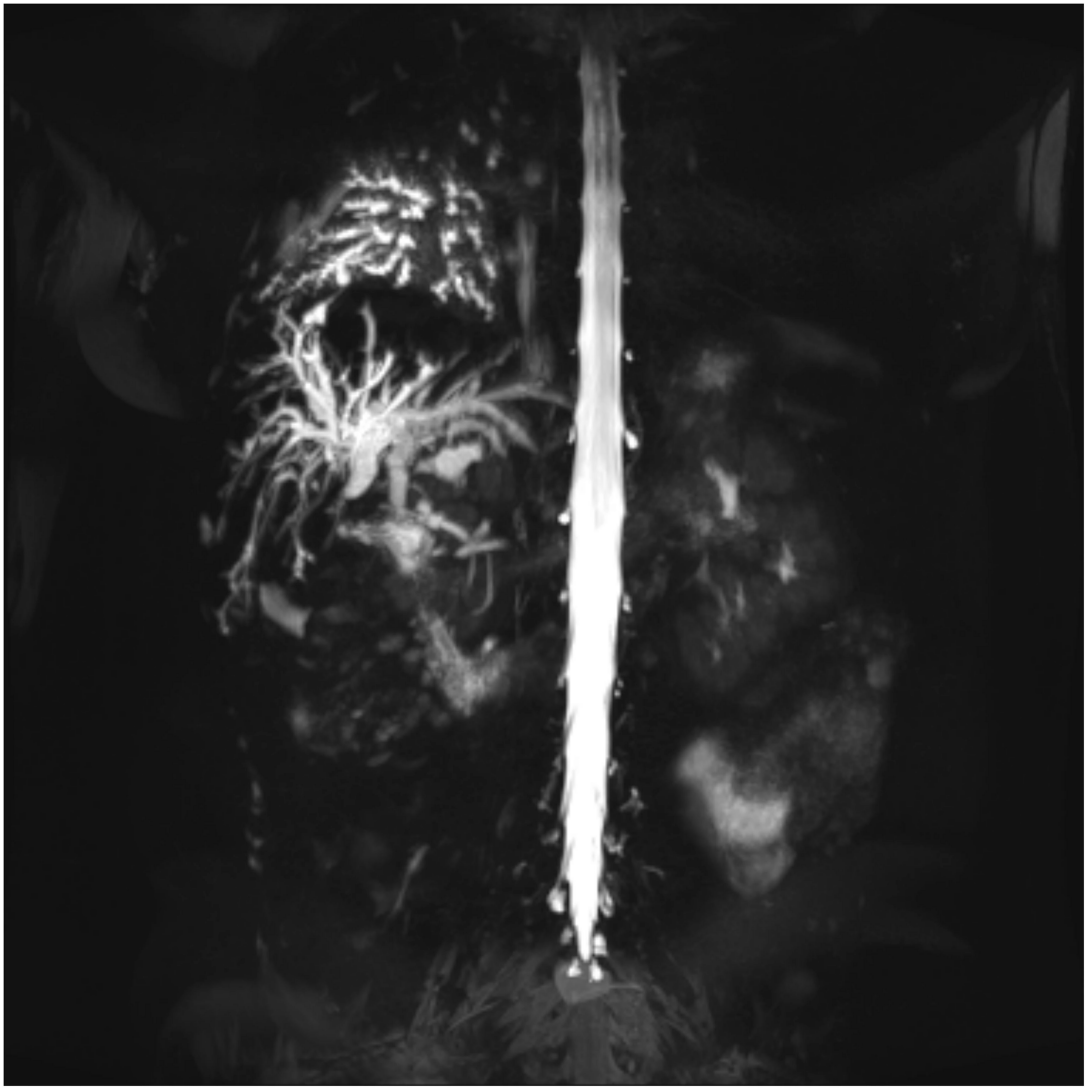
Cholangiography—MRI showing the fistulous path.

Magnetic resonance cholangiography confirmed that bile duct stenosis and the interconnection of the bile duct between the posterior region of the liver and the right lower bronchi originated after the sequelae of cholecystectomy (Figure 3).

The surgical team performed an external biliary bypass, and the patient presented clinical improvement and was discharged 3 days after the procedure. At this time, laboratory tests showed decreased levels of total bilirubin = 3.1 mg/dL, conjugated bilirubin = 2.7 mg/dL, AST = 40 U/L, ALT = 46 U/L, GGT = 503 U/L and AP = 593 U/L.

**Figure 4:**
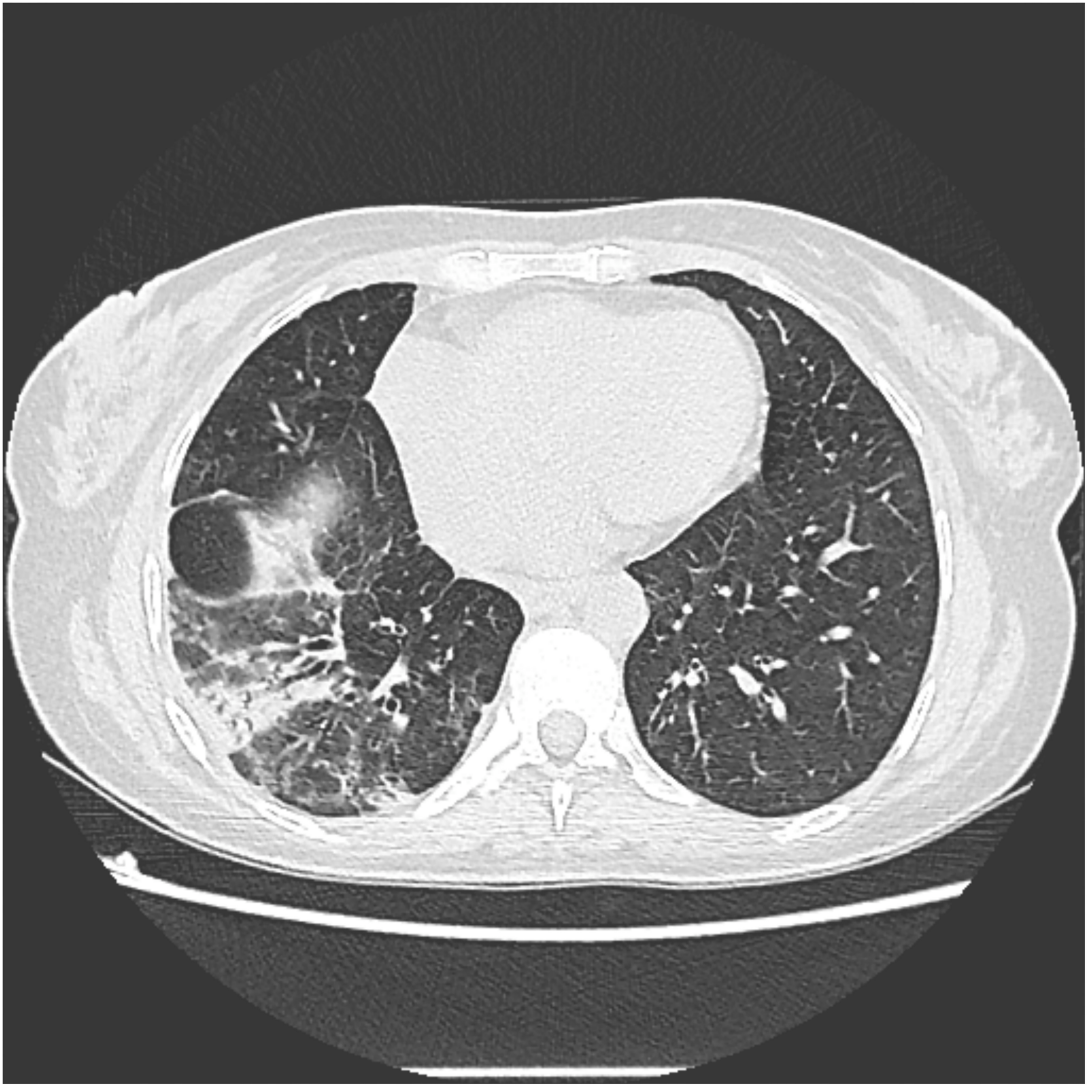
Slice of CT scan showing parenchymal fibrosis and bronchiectasis in the right lower lobe.

She was re-evaluated 1 month later by the surgical team who decided to continue with the external biliary bypass regarding the liver cirrhosis. We performed a new chest CT scan 4 months after surgery, which demonstrated partial resolution of the inflammatory process. However, it presented a sequelae bronchiectasis with parenchymal fibrosis (Figure 4). At this time, the patient did not present respiratory symptoms or expectoration.

## Discussion

The physiopathology of BBF is related to injury of the adjacent lung or pleural space by erosion of the diaphragm. Consequently, the expansion of a subphrenic infected biloma with a concomitant liver and a direct diaphragmatic injury are described in cases of tumors, or in postoperative or post-ablation cases (biliary stenosis/lithiasis). In postoperative iatrogenic stenosis, such as reported in this case, the bile duct obstruction may lead to cholangitis, intrahepatic abscess, and rupture of abscess into the pleural space or bronchi when the complication is associated with adhesions. This fact explains the preferential location of bronchobiliary fistulas on the right side of the chest. However, some exceptional cases of bronchobiliary fistulas located on the left side have also been described in literature due to invasive liver disease, tumor metastases, or infectious diseases with direct involvement of the diaphragm and pleural space (8–10).

A systematic review of 68 cases published between January 1980 and August 2010 showed that the main clinical manifestation was bilioptysis, which was present in all 68 reports. The volume of biliary sputum ranged from 200 to 600 ml and reached a maximum of 1.2 L daily. More than half the patients presented with fever; other clinical features were jaundice and respiratory symptoms, such as irritating cough and dyspnea (2). Chest and right upper quadrant abdominal pain can occur (11). Some patients with a chronic course may develop potentially debilitating and life-threatening conditions such as bronchiectasis, hepatic lung disease, portal hypertension, and anemia (7, 11). Our patient developed a chronic cough, dyspnea, and bilioptysis, associated with unexpected cyclic jaundice.

One diagnostic criterion proposed is imaging methods proving BBF or bile accumulations in the pleura (3, 7, 12). The most sensitive diagnostic methods are endoscopic retrograde cholangiopancreatography, percutaneous transhepatic-cholangiography, and a fistulogram of the biliocutaneus fistula (13). Correspondingly, a CT scan can be helpful for detecting air in the biliary tree, underlying hepatic abscess or hepatic biloma, pleural effusion, or pulmonary consolidation, but it might fail to demonstrate a fistula tract (11, 13). Contrast-enhanced cholangiography-MRI can identify fistulous tracts.

The other criterion is the documentation of bilirubin in sputum or BAL. However, false biliary ptyalism has been described in patients with sickle cell disease and hemolytic crisis and should be considered a confounding factor. In patients without such conditions, the presence of bile in the sputum is defined as pathognomonic BBF (14). There is no standardized method of analyzing sputum for bilirubin since it is not a routine lab test. During a bronchoscopy exam, bile in the lavage fluid may not be located. Even so, biliary pneumonia and BBF diagnosis can be made when brown bilious crystals surrounded by neutrophils are found in the transbronchial biopsy (13, 15). Bilirubin crystallization in bronchoalveolar lavage fluid can also be proof of bilirubin in the airway (16, 17). Similarly, finding bilirubin in the pleural fluid can also be used to diagnose biliopleural fistula (3, 13).

Both criteria were fulfilled in our case: bilirubin was identified in the sputum by indirect methods, and a fistulous path between the biliary tract and the bronchial tree was detected in imaging.

The BBF treatment is controversial in the literature. The use of somatostatin and its analogs has been proposed to reduce gastrointestinal secretion, but this has not been effective as a monotherapy (13). Surgical intervention is the most common treatment.

Surgical treatment is a conservative method for chronic fistulas associated with respiratory failure or sepsis, or when BBF is secondary to tumors, biliary obstruction, and trauma. Literature shows that most patients (41.7%) are treated by surgical procedures, including pulmonary lobectomy, resection, and correction of the fistula tract in the diaphragm; hepatolobectomy; hepatic enterostomy; and abscess drainage alone or in combination (3, 13).

The first stage is to perform external biliary drainage by percutaneous or surgical drainage of subphrenic abscess and/or direct percutaneous drainage of the intrahepatic biliary tract. The second stage is to perform treatment of the underlying cause. In patients with biliary obstruction, the priority is to treat the biliary disease (8).

Less-invasive procedures tended to be employed in treating BBF, and these include endoscopic retrograde biliary drainage, endoscopic nasobiliary drainage, and endoscopic stone extraction. External biliary drainage by percutaneous ultrasound or CT scan can drain the subdiaphragmatic or intrahepatic abscess. Bronchoscopy can lead to fistula embolization. However, control of the inflammatory process can contribute to the success or failure of these methods (7, 13). External biliary drainage was performed in our case as a bridge for later definitive surgical intervention. After the procedure, cough and fever symptoms were immediately relieved, demonstrating satisfactory lung parenchyma recovery at that moment.

A transthoracic approach should be considered for rare situations in which patients maintain irreversible pulmonary and bronchial impairment or for those with traumatic bronchobiliary fistula without biliary obstruction. In this situation, pleural decortication and diaphragmatic disruption repair are recommended (8, 13).

In conclusion, although BBF is a rare condition when bilioptysis is suspected, a diagnostic investigation should be initiated. Our case study proposes two criteria for diagnosis: an imaging exam demonstrating the fistula path and a proof of bilirubin in sputum or BAL. Once diagnosed and the mechanism clarified, surgical correction is indicated. Less invasive methods are preferred over conservative methods, which are reserved for more complex cases or when treatment fails. However, individualized treatment should be emphasized for a successful outcome.

## Data availability statement

The original contributions presented in the study are included in the article/supplementary material, further inquiries can be directed to the corresponding author/s.

## Ethics statement

The studies involving human participants were reviewed and approved by Hospital das Clínicas of Botucatu Medical School. The patients/participants provided their written informed consent to participate in this study. Written informed consent was obtained from the individual(s) for the publication of any potentially identifiable images or data included in this article.

## Author contributions

All authors listed have made a substantial, direct, and intellectual contribution to the work and approved it for publication.
